# Machine learning for automated cause-of-death classification from 2021 to 2022 in Korea: development and validation of an ICD-10 prediction model

**DOI:** 10.12771/emj.2025.00675

**Published:** 2025-07-28

**Authors:** Seokmin Lee, Gyeongmin Im

**Affiliations:** Statistics Research Institute, Statistics Korea, Daejeon, Korea

**Keywords:** Cause of death, Deep learning, International Classification of Diseases, Korean Standard Classification of Diseases-8th Revision

## Abstract

**Purpose:**

This study evaluated the feasibility and performance of a deep learning approach utilizing the Korean Medical BERT (KM-BERT) model for the automated classification of underlying causes of death within national mortality statistics. It aimed to assess predictive accuracy throughout the cause-of-death coding workflow and to identify limitations and opportunities for further artificial intelligence (AI) integration.

**Methods:**

We performed a retrospective prediction study using 693,587 death certificates issued in Korea between January 2021 and December 2022. Free-text fields for immediate, antecedent, and contributory causes were concatenated and fine-tuned with KM-BERT. Three classification models were developed: (1) final underlying cause prediction (International Classification of Diseases, 10th Revision [ICD-10] code) from certificate inputs, (2) tentative underlying cause selection based on ICD-10 Volume 2 rules, and (3) classification of individual cause-of-death entries. Models were trained and validated using 2021 data (80% training, 20% validation) and evaluated on 2022 data. Performance metrics included overall accuracy, weighted F1 score, and macro F1 score.

**Results:**

On 306,898 certificates from 2022, the final cause model achieved 62.65% accuracy (F1-weighted, 0.5940; F1-macro, 0.1503). The tentative cause model demonstrated 95.35% accuracy (F1-weighted, 0.9516; F1-macro, 0.4996). The individual entry model yielded 79.51% accuracy (F1-weighted, 0.7741; F1-macro, 0.9250). Error analysis indicated reduced reliability for rare diseases and for specific ICD chapters, which require supplementary administrative data.

**Conclusion:**

Despite strong performance in mapping free-text inputs and selecting tentative underlying causes, there remains a need for improved data quality, administrative record integration, and model refinement. A systematic, long-term approach is essential for the broad adoption of AI in mortality statistics.

## Introduction

### Background

In 2023, the number of deaths in South Korea reached 352,511, representing a 32.4% increase since 2013. As one of the fastest-aging societies, South Korea is projected to experience continued surges in mortality, with more than 500,000 deaths expected by 2038, according to 2022 population projections [1]. These trends present major challenges for compiling cause-of-death statistics, which are vital for public health and demographic policy and necessitate improvements in data collection and analysis workflows.

Statistics Korea compiles these statistics based on physician-issued medical death certificates and family-reported death registrations. Causes are recorded in accordance with World Health Organization (WHO) guidelines, including the immediate cause, up to 3 antecedent causes, and other contributory physical conditions. Statistical reporting focuses on the underlying cause. To maintain international comparability, WHO specifies rules for underlying cause selection in the International Classification of Diseases, 10th Revision (ICD-10) Volume 2 [2].

However, physicians often lack sufficient information when completing death certificates. To address this, Statistics Korea supplements its data with 22 types of administrative records, such as national health insurance data, cancer registries, police investigations, autopsy reports, and infectious disease notifications. Cause-of-death coders, certified both internationally and domestically, review these materials to finalize the underlying cause. As mortality rises, the workload for these professionals also increases, increasing the risk of errors.

Meanwhile, artificial intelligence (AI) has begun to supplant human judgment in various sectors. Its potential to alleviate the growing burden of mortality statistics makes it a promising tool. This study explores the feasibility of leveraging AI for the compilation of cause-of-death data.

### Objectives

This study investigates the ways in which AI can support the compilation of cause-of-death statistics and identifies its current limitations. Maintaining statistical quality amid rising mortality presents a complex challenge; thus, early assessment of AI’s effectiveness is crucial. By analyzing real-world applications, we assess the potential of AI and propose strategies for its integration into future workflows.

## Methods

### Ethics statement

This study involved analysis of publicly available data; therefore, approval from an institutional review board and informed consent were not required.

### Study design

This prediction study was conducted using publicly available data from Statistics Korea. The study design adheres to the TRIPOD-AI reporting guidelines for deep learning applications in medical research (development or prediction), available at https://www.tripod-statement.org/.

### Setting

The study setting was the nationwide death registration system of the Republic of Korea. Data were obtained from national death certificates issued between January 1, 2021, and December 31, 2022, totaling 319,198 certificates in 2021 and 374,389 in 2022. The dataset thus represents a general population setting, covering deaths from all regions of Korea (urban and rural) and all levels of care, including hospital deaths with detailed medical information and home deaths that may have less detail.

### Participants

We effectively included the entire population of deaths in Korea during 2021–2022 for which a cause-of-death code was assigned.

### Data source

Raw data were extracted from the Causes of Death Statistics database maintained by Statistics Korea. The study utilized 693,587 death certificate records (319,198 from 2021 and 374,389 from 2022), as coded by Statistics Korea, along with text input data from the underlying cause-of-death selection system (693,195 records) submitted by physicians or officers. Only records with matching case numbers across both datasets were selected and used as training data.

**Fig. 1.**
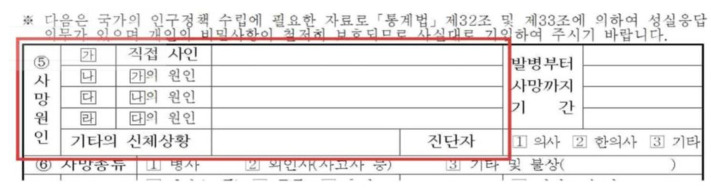
Cause-of-death section on Korean death certificates.

The cause-of-death section on Korean death certificates typically includes multiple entries: an immediate cause, intermediate causes, the underlying cause, and other contributing conditions (covering Part I and Part II of the WHO death certificate format) (Fig. 1).

However, coding rules require assignment of a single underlying cause-of-death code based on all this information. For modeling purposes, we concatenated the text from all cause-of-death fields to provide the model with full context. Specifically, we constructed a narrative summary by joining the entries in order—from immediate cause down to underlying cause, along with any other significant conditions—separated by punctuation. This approach mirrors how human coders review the entire certificate to determine the underlying cause.

### Data preprocessing

**Fig. 2.**
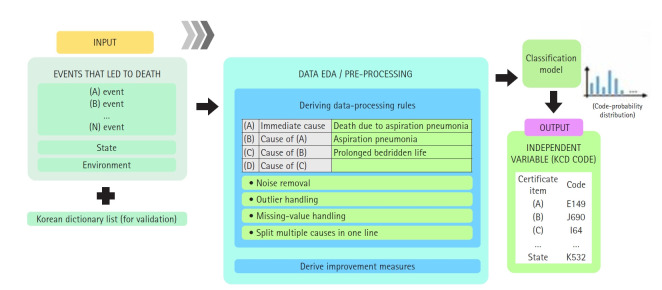
Information on mortality statistics data: inputs (cause of death), preprocessing, and outputs (automated candidate underlying causes).

Detailed preprocessing steps are presented in Supplement 1. Information on mortality statistics data—including inputs (cause of death), preprocessing, and outputs (automated candidate underlying causes)—is summarized in a diagram (Fig. 2)

### Outcome variables/predictors

The outcome of interest was the underlying cause-of-death code, as defined by ICD-10, for each record. Thus, the model predicts a single label (the ICD-10 code) for each death certificate. The predictor variable is the textual content of the death certificate.

### Bias

There was no bias in data collection or analysis, as all target data were included.

### Study size

The study included the entire population of the Republic of Korea. No sample size estimation was necessary. Records with missing data were excluded from the analysis. Any death certificate lacking cause text was removed from the dataset before analysis, ensuring that the models did not encounter missing inputs.

### Machine learning models

The Korean Medical BERT (KM-BERT) language model [3], a medical-domain pretrained BERT for Korean, was employed for natural language processing. KM-BERT is trained on over 60 million sentences and 1.16 billion Korean medical terms. Fine-tuning was conducted to achieve robust performance in medical term classification. Four models were trained and evaluated as described below:

#### Experiment 1: Development of a predictive model for the final underlying cause of death (8th Korean Standard Classification of Diseases and Causes of Death [KCD-8] code) using death certificate input fields

• Data source: All death certificate records with matching case numbers (N=693,194).

• Model task: Directly predict the final underlying cause (KCD-8 code) from the input items on each death certificate.

• Objective: Quantify the rate at which forensic examiners revise the model’s predicted classification for each cause-of-death category.

#### Experiment 2: Development of a baseline model for evaluating classification quality and similarity analysis of the automated underlying cause system

• Data source: A curated dataset of 310,034 death certificate records in which the automated underlying cause and the final underlying cause coincide.

• Model task: Using this curated dataset, a baseline classifier was trained to replicate the outputs produced by the automated underlying cause system.

• Objective: To validate the classification quality and analyze similarity between the model’s predictions and the existing automated underlying cause results (with a particular focus on disease categories).

#### Experiment 3: Development of a knowledge-based classification model using only established resources

• Data source: Unstructured text fields from death certificates.

• Training resources: (1) An existing Korean language dictionary containing 74,123 entries; (2) The KCD-8 (based on ICD-10) master file, which has 89,584 Korean and English terminology entries (Supplement 2).

• Model task: Predict the classification code for each cause of death input field using only these curated vocabularies and domain knowledge.

• Objective: Evaluate the expected performance of a system built exclusively on pre-existing linguistic resources and domain expertise, without additional training examples.

### Evaluation metrics

Accuracy, F1-macro, F1-weight, and loss were measured for each model.

#### Accuracy

The proportion of total predictions that the model classifies correctly. It is calculated as accuracy=[number of correct predictions]/[total number of predictions].

#### F1 macro

The arithmetic mean of the F1 scores computed independently for each class, giving equal weight to all classes regardless of their frequency. This metric reflects the model’s balanced performance across both common and rare classes.

#### F1 weighted

The weighted average of per-class F1 scores, where each class’s F1 is weighted by its support (the number of actual instances). This metric emphasizes performance on more prevalent classes, while still accounting for minority classes.

### Statistical methods

Aside from the machine learning evaluation metrics described above, traditional statistical hypothesis testing for effect sizes or risk factors was not employed. The primary statistic of interest was model accuracy.

## Results

### Final underlying cause prediction (Experiment 1)

To determine the degree to which AI predictions of the underlying cause of death align with the actual causes, we trained the model solely on death certificates. We compared the underlying cause predicted by AI with the official underlying cause of death, which is determined using both death certificates and multiple administrative data sources. Of the 317,356 death certificates issued in 2021, a total of 140,080 records were used for training and validation. We then evaluated and compared results using 306,898 cases reported in 2022. The optimal classification model was reached at 300 steps (approximately 50 epochs). Upon evaluation of the 306,898 records from 2022, the model achieved a consistency rate of 62.65%. Approximately 37.35% of cases required modification, primarily due to the influence of external causes of death (Table 1).

### Tentative underlying cause prediction (Experiment 2)

To evaluate the intermediate steps in generating cause-of-death statistics, we compared the tentative underlying cause—derived without administrative data—to the official results. Statistics Korea uses a term mapping table to automatically convert conditions written in Korean on death certificates into codes, then selects a tentative underlying cause according to WHO ICD-10 Vol. 2 guidelines. Measuring AI’s ability to replicate this process may help reduce the burden of mapping table management.

**Fig. 3.**
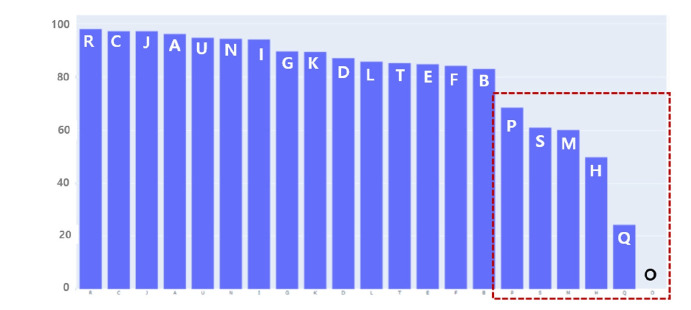
Accuracy of artificial intelligence’s prediction of the tentative underlying cause of death by major sections.

A total of 310,034 refined datasets, in which the results before and after administrative data supplementation were identical, were used for training. The baseline model was trained using this curated data, and KM-BERT was employed as the language model. Optimal learning was achieved at 270 steps (approximately 90 epochs). Evaluation demonstrated high accuracy at 95.35% (Table 1). However, detailed results by classification item revealed areas of weakness in AI prediction. Of the 1,026 codes evaluated, 291 codes (e.g., E272, I7110, C740, C109) were highly reliable (F1-score ≥0.9), while 410 codes (e.g., L892, A1830, R578, R609) were unreliable (F1-score=0). Unreliable results were particularly prominent for codes in “P. Prenatal and postnatal conditions,” “S. Injury and poisoning,” “M. Musculoskeletal diseases,” “H. Eye and ear diseases,” “Q. Congenital malformations,” and “O. Pregnancy, childbirth, and postpartum diseases,” all of which require supplemental administrative data. These findings indicate that specific disease categories require further model revision (Fig. 3).

The number of misclassified causes of death was 18,116, with the majority being rare diseases such as anencephaly or cholera, which lacked sufficient training data. Many subcategories were also underrepresented, underscoring the need for expanded case data tailored to each classification purpose.

A typical error type occurred when frequently recorded causes of death, such as sepsis, were involved. In these cases, AI tended to predict categories for which it had more abundant training data. For example, when both sepsis and myelitis were listed, the model predicted M46.99 (unspecified inflammatory spondylopathy, unspecified site) instead of the more specific M46.59 (other infectious spondylopathy, unspecified site). This misclassification reflects the high frequency with which sepsis is recorded on death certificates for unspecified inflammatory spondylopathy.

### Cause-of-death coding on death certificates (Experiment 3)

The accuracy of ICD-10 coding of causes of death described in natural language on death certificates was compared between artificial intelligence and term mapping table methods. Training data included the ICD-10 code list and the term mapping table used by Statistics Korea. The optimally fine-tuned classification model was learned at 1,550 steps (approximately 50 epochs). Evaluation showed that the AI coding accuracy was relatively low at 79.51%. However, the F1-Macro score was very high at 0.925, indicating strong agreement for items where prediction results were produced (Table 1).

## Discussion

### Key results

The process of producing cause-of-death statistics is primarily divided into 3 stages: (1) coding of natural language on death certificates, (2) selection of the tentative underlying cause of death according to ICD-10 Volume 2, and (3) modification of the final underlying cause of death based on administrative data. To determine what AI can predict at each step, we analyzed both the official statistical outcomes and AI prediction results. Experimentally, the model achieved 62.65% accuracy for the final underlying cause of death, 95.35% accuracy for the tentative underlying cause, and 79.51% coding accuracy for the cause of death [3].

### Interpretation

Even for provisional causes of death where overall accuracy was high, unreliable results were observed for specific diseases. Understanding the overall volume of deaths is important, but so is managing trends for detailed causes and rare diseases. Thus, even rare diseases or deaths with low occurrence must be classified accurately. In this regard, our experiment highlights considerations relevant to the introduction of AI in this field.

### AI misclassification cases

There are 3 major types of errors that AI can make when generating cause-of-death statistics. First, AI can confuse symptoms commonly listed on death certificates. For instance, sepsis often appears as a symptom secondary to various causes of death. When sepsis, spondylitis, and pneumonia are all present, the case should be classified as M46.59 (other infectious spinal diseases), yet it is frequently misclassified as M46.99 (unspecified inflammatory spinal diseases), a common error.

Second, errors may arise from insufficient understanding of causal relationships in disease coding. For example, diabetic renal failure should be classified as E14.28 (unspecified diabetes with renal complications), but the AI instead predicts E14.9 (unspecified diabetes without complications).

Third, errors occur when the AI lacks a nuanced understanding of natural language on death certificates. For instance, when the certificate states “동맥 관 개방,” it should be classified as Q25.0 (patent ductus arteriosus), but is instead classified as Q21.4 (aortopulmonary septal defect). Such errors often result from limited training data or ambiguity in language processing during AI calculation.

### Limitations of applying AI to cause-of-death statistics

The classification for cause of death includes approximately 18,000 categories, far more detailed than the industrial classification system of about 1,200 categories. Accurate classification is crucial, not only for common causes but also for rare or infectious diseases, as these statistics are fundamental for public health policy. Therefore, a higher standard of accuracy is required compared to other statistical domains.

Considering these characteristics, applying AI to cause-of-death statistics poses significant challenges in predicting specific diseases or rare cases, since traditional modeling procedures may be inadequate. AI performance depends greatly on the diversity and volume of training data. Misclassification is particularly likely for rare or critical diseases with limited data.

To achieve successful AI application in this area, methods for correcting errors in specific cases must be considered. Supplementation with administrative data is also essential, and future AI models will require technology capable of verifying and integrating large-scale administrative records.

### Directions of development for AI applications for cause-of-death statistics

Accurate prediction of specific diseases may benefit from a hybrid approach that combines case-based inference with established knowledge construction techniques, such as mapping tables or information retrieval methods. Additional experiments and advanced research are needed to build fine-tuned models using ensemble methods—leveraging existing machine learning algorithms capable of handling small sample sizes, large language models with extensive parameters, or specialized models focused on diseases and causes of death.

Furthermore, because cause-of-death statistics significantly impact public health policy, a long-term, systematic review of AI introduction is needed for each procedural step. Importantly, if the quality of death certificates—serving as the fundamental data source—does not improve, both the input quality and completeness of AI training data will suffer. It is therefore critical that death certificates are completed thoroughly and accurately.

### Conclusion

The growing proportion of older adults in the population is causing a sharp rise in the number of deaths, which in turn may affect the quality of cause-of-death statistics. Statistics Korea utilizes an automated program to derive tentative causes of death from information listed on death certificates, followed by review and revision of the final cause based on administrative data. As the burden of statistical work increases, process quality may be compromised. Thus, integrating AI into the workflow represents a substantial opportunity for improvement.

However, because cause-of-death statistics involve more categories, higher complexity, and greater societal impact than other statistical fields, any changes in workflow must be accompanied by continuous, long-term review and analysis. In this study, we conducted a focused analysis using AI models trained on 2 years of data, confirming accuracy at each stage and identifying AI’s limitations. This work is expected to lay the foundation for more effective AI applications in this area, ultimately supporting a healthier and safer society by enhancing the quality of cause-of-death statistics.

## Figures and Tables

**Table 1.** Summary of the validation and evaluation protocols for Experiments 1–4

Experiment	Input variables (X)	Output variables (Y)	Training/validation data	Evaluation data (comparison)	Accuracy	F1 score (weighted)	F1 score (macro)
1	Cause of death input information (5 items): direct cause, primary antecedent cause, secondary antecedent cause, tertiary antecedent cause, physiological condition	Final underlying cause classification code	2021 published data: input and classification codes (training: validation=8:2)	2022 published final underlying cause classification codes (entire dataset)	0.6265	0.594	0.1503
2	Cause of death input information (5 items): direct cause, primary antecedent cause, secondary antecedent cause, tertiary antecedent cause, physiological condition	Final underlying cause classification code	2021 published data (curated subset where automatic underlying cause=final underlying cause): input and classification codes	2022 published automatic underlying cause classification codes	0.9535	0.9516	0.4996
3	Individual cause per record (single item)	Individual cause classification code	Existing Korean terminology dictionary+ICD-10 database master file	Cause-of-death selection system (simulation) results (20% test split) classification codes	0.7951	0.7741	0.925

ICD-10, International Classification of Diseases, 10th Revision.

## Supplementary materials

Supplementary files are available from Harvard Dataverse: https://doi.org/10.7910/DVN/VT7IUV

Supplement 1. Data preprocessing and description of utilized data.

Supplement 2. KCD-8 DB master file.
