# Supplementary material for: Machine learning for automated cause-of-death classification from 2021 to 2022 in Korea: development and validation of an ICD-10 prediction model
Source: Ewha Med J. 2025 Jul 28;48(3):e45. doi: 10.12771/emj.2025.00675 (PMC12362283; doi:10.12771/emj.2025.00675)
Supplement: Supplementary file 1 — Supplement 1. Data preprocessing and description of utilized data. [file emj-2025-00675-Supplementary-1.pdf]

Supplement 1. Data preprocessing and description of utilized data

Preprocessing

In relation to the task of coding the disease conditions, data preprocessing processes such as lowest-level code validity verification and separation of 2 or more disease conditions per line of the death certificate were performed. The code validity verification was confirmed using the 8th Korean Standard Classification of Diseases and Causes of Death (KCD-8) master file, which is a Korean classification based on the International Classification of Diseases, 10th Revision (Supplement 2). Separation of 2 or more disease conditions per line of the death certificate was separated by commas and word-by-word analysis.

Training, verification, and evaluation data

Final underlying cause prediction> (Experiment 1)

- Extract 693,194 data linked to the table for underlying cause of death among 693,587 death certificates.
- Excluding 68,940 cases where the final underlying cause was coded as unknown or not in the classification system, 624,254 cases were used.
- After that step, 306,898 cases in 2022 were used as evaluation data, and 317,356 cases in 2021 were used as learning and verification data.
- Added 51,090 data with 1 label for stratified extraction of learning and verification data.
- Afterwards, 80% (294,756 cases) were separated into training data and 20% (73,690 cases) into verification data, and then duplicates were removed.
- Finally, 106,089 cases of training data, 33,991 cases of verification data, evaluation data 306,898 cases selected.

Tentative underlying cause prediction (Experiment 2)

- Extracted 310,034 cases with the same tentative cause and final cause of death out of a total of 693,587 cases.
- Excluding 902 cases where the final cause was coded as unknown or not in the classification system.
- Separated 150,746 cases in 2022 into evaluation data and 158,386 cases in 2021 into training and verification data.
- Added 1,836 data with 1 label for stratified extraction of learning and verification data.
- After that, 80% (128,177 cases) were separated into learning data and 20% (32,045 cases) into verification data, and then duplicates were removed.
- Finally, 48,723 cases of training data, 15,077 cases of verification data, and 150,746 cases of evaluation data were selected.

Coding of cause of death on death certificate (Experiment 3)

- The training and verification data were selected as 163,707 cases, which were integrated from 89,584 KCD-8 master miles and 74,123 cases of the mapping table of the cause of death selection system.
- Of these, 80% (130,966 cases) were used as training data, and 20% (32,741 cases) were used as verification data.
